# Supplementary material for: Cellular insights of beech leaf disease reveal abnormal ectopic cell division of symptomatic interveinal leaf areas
Source: PLoS One. 2023 Oct 5;18(10):e0292588. doi: 10.1371/journal.pone.0292588 (PMC10553357; doi:10.1371/journal.pone.0292588)
Supplement: S1 Fig — (PDF) [file pone.0292588.s008.pdf]

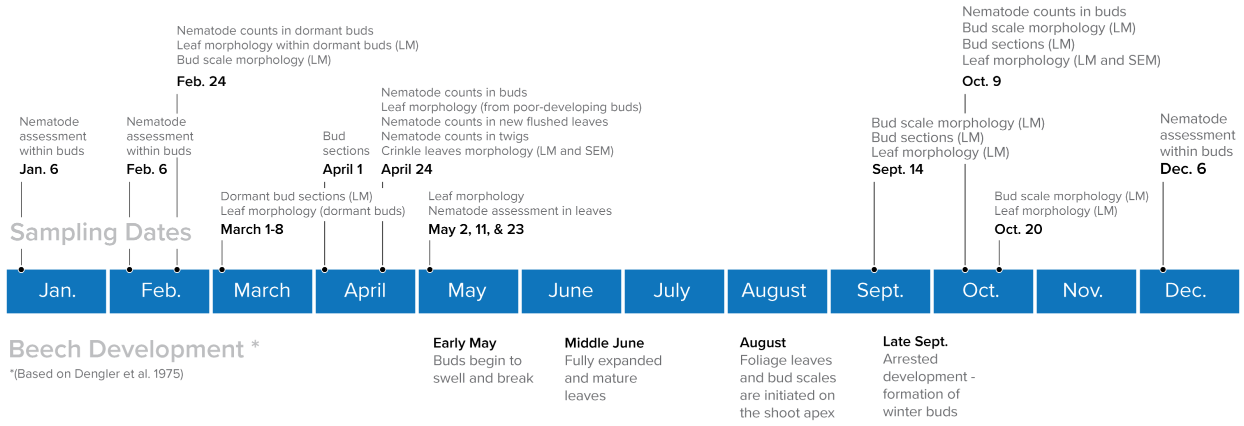

S1 Fig. Graphic representation of sampling dates and beech bud and leaf main developmental stages.
