# Supplementary material for: Cellular insights of beech leaf disease reveal abnormal ectopic cell division of symptomatic interveinal leaf areas
Source: PLoS One. 2023 Oct 5;18(10):e0292588. doi: 10.1371/journal.pone.0292588 (PMC10553357; doi:10.1371/journal.pone.0292588)
Supplement: S2 Fig — A total of 60 buds were collected randomly at the beginning of the fall from beech leaf disease symptomatic trees. (A) A representative set of beech buds ready to be processed for nematode extraction and morphological analysis. (B) Eggs in different developmental stages. (C-E) Juvenile stages. (F-G) Mature sexual adults. F: male; G: female. (H) Putative asexual female. Sclerotized, cuticularized anal openings were at times more difficult to observe in asexual females than in sexual, reproductive females. However, the strongly sclerotized stylet was always distinctly robust, and longer in sexually reproductive females than in asexual females [10.0 ± 0.0 (10.0–10.0) vs 7.4 ± 0.2 (7.0–7.5)], with a thicker, well-developed stylet and stylet knobs vs a thin stylet with weak, almost absent knobs. In addition, the location of the vulva on the body (V %) was higher in sexually reproductive females than in asexual females [80.1 ± 0.5 (79.6–81.0) vs 77.6 ±1.1 (75.0–79.1)]; and the tail length was smaller, with a mostly thorn-like terminus in sexually reproductive females [47.8 ± 8.9 (39.0–62.0) vs 59.9 ±7.1 (50.0–70.0)] with a long, pointed terminus in asexual females. The asexual females have longer esophageal glands, and longer, filiform tail. s: stylet; v: vulva. Scale bars: 20 μm. (PDF) [file pone.0292588.s009.pdf]

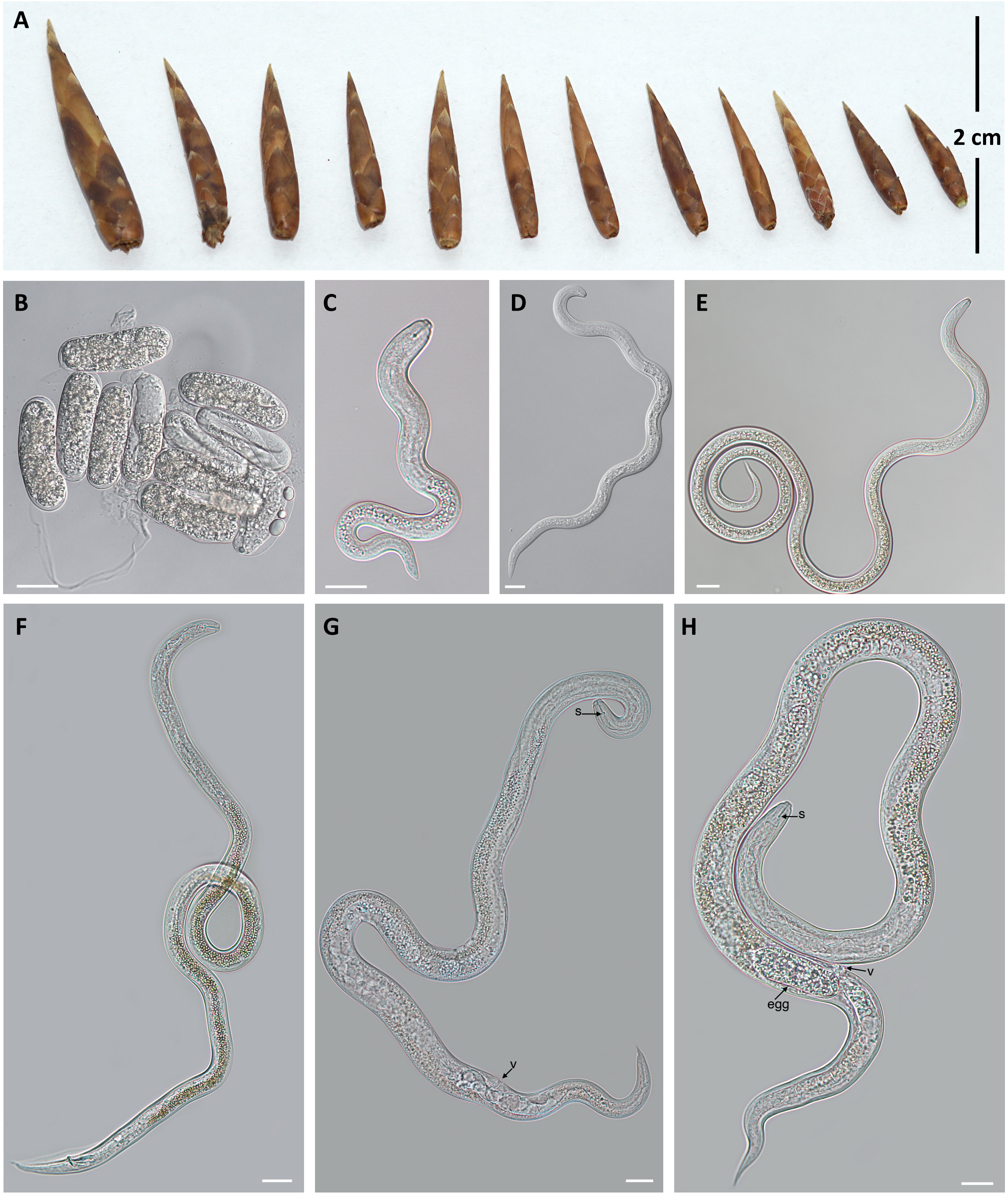

S2 Fig. Beech leaf disease nematode, *Litylenchus crenatae* subsp. *mccannii*, associated with naturally infected beech buds. A total of 60 buds were collected randomly at the beginning of the fall from beech leaf disease symptomatic trees. **(A)** A representative set of beech buds ready to be processed for nematode extraction and morphological analysis. **(B)** Eggs in different developmental stages. **(C-E)** Juvenile stages. **(F-G)** Mature sexual adults. F: male; G: female. **(H)** Putative asexual female. Sclerotized, cuticularized anal openings were at times more difficult to observe in asexual females than in sexual, reproductive females. However, the strongly sclerotized stylet was always distinctly robust, and longer in sexually reproductive females than in asexual females [10.0 ± 0.0 (10.0-10.0) vs 7.4 ± 0.2 (7.0 -7.5)], with a thicker, well-developed stylet and stylet knobs vs a thin stylet with weak, almost absent knobs. In addition, the location of the vulva on the body (V %) was higher in sexually reproductive females than in asexual females [80.1 ± 0.5 (79.6-81.0) vs 77.6 ±1.1 (75.0 -79.1)]; and the tail length was smaller, with a mostly thorn-like terminus in sexually reproductive females [47.8 ± 8.9 (39.0-62.0) vs 59.9 ±7.1 (50.0-70.0)] with a long, pointed terminus in asexual females. The asexual females have longer esophageal glands, and longer, filiform tail. s: stylet; v: vulva. Scale bars: 20 µm.
