# Supplementary material for: Cellular insights of beech leaf disease reveal abnormal ectopic cell division of symptomatic interveinal leaf areas
Source: PLoS One. 2023 Oct 5;18(10):e0292588. doi: 10.1371/journal.pone.0292588 (PMC10553357; doi:10.1371/journal.pone.0292588)
Supplement: S3 Fig — (A-C) Nematode-infected bud scales showing different levels of cell hypertrophy within scales of the same bud (arrows point out nematodes). (D) Clusters of eggs and motile stages found in-between the bud scales. Scale bars: 50 μm. (PDF) [file pone.0292588.s010.pdf]

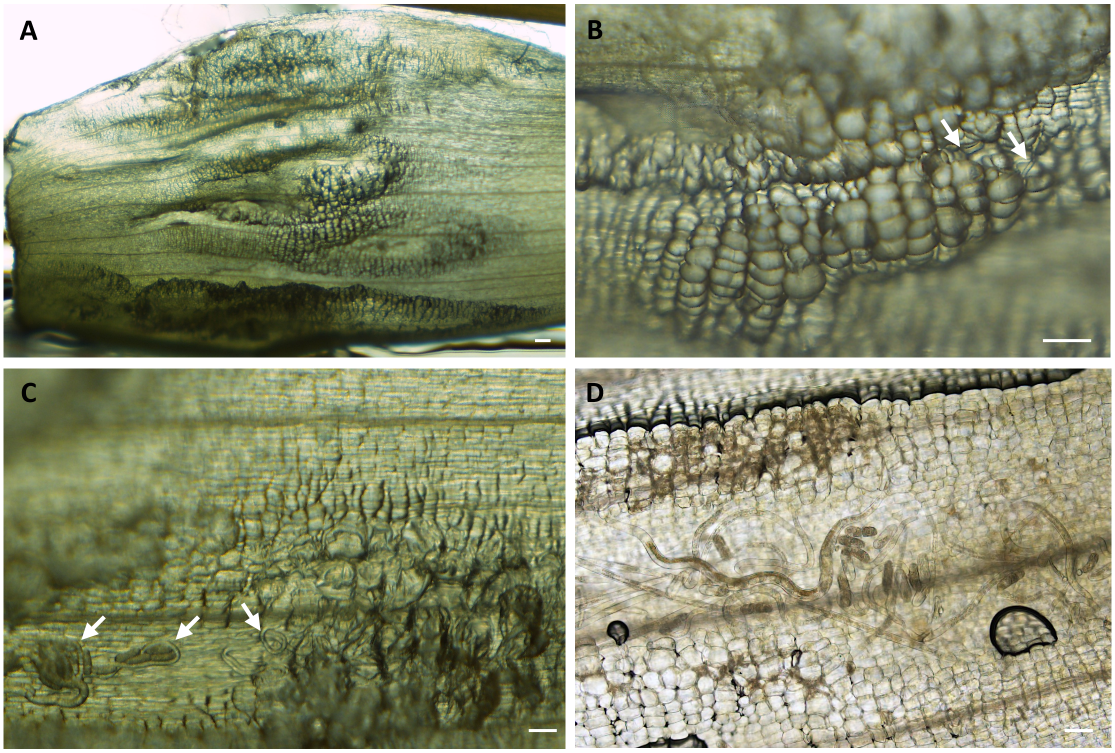

S3 Fig. Examples of enlarged cell associated with nematode-infected (*Litylenchus crenatae* subsp. *mccannii*) bud scales. (A-C) Nematode-infected bud scales showing different levels of cell hypertrophy within scales of the same bud (arrows point out nematodes). **(D)** Clusters of eggs and motile stages found in-between the bud scales. Scale bars: 50 µm.
