# Supplementary material for: Cellular insights of beech leaf disease reveal abnormal ectopic cell division of symptomatic interveinal leaf areas
Source: PLoS One. 2023 Oct 5;18(10):e0292588. doi: 10.1371/journal.pone.0292588 (PMC10553357; doi:10.1371/journal.pone.0292588)
Supplement: S4 Fig — (A) Aggregated nematodes (arrow) were often detected between the bud scales. (B) Representative image of nematodes found associated with the buds at the beginning of winter. (C-F) Dead nematodes found within the buds; (C-D) eggs and (E-F) adult females. (G-I) Active juvenile nematodes with characteristic dark intestines that are filled with numerous lipid droplets. Immature females are the most common and active stage (I). Scale bars: 200 μm (A, B); 20 μm (C-I). (PDF) [file pone.0292588.s011.pdf]

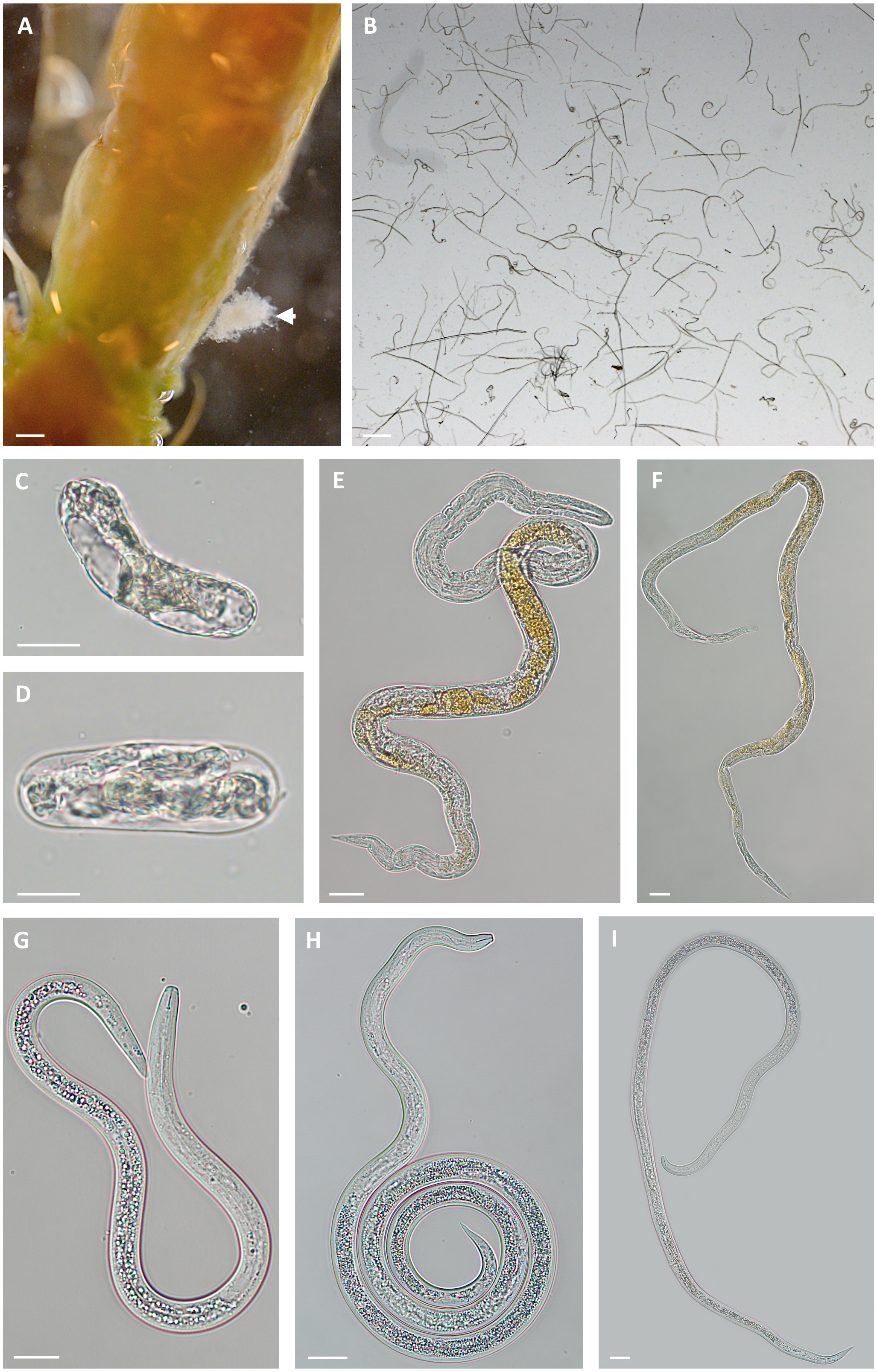


**S4 Fig. Nematodes (*Litylenchus crenatae* subsp. *mccannii*) associated with naturally infected beech (*Fagus grandifolia*) buds collected in the beginning of the winter. (A)** Aggregated nematodes (arrow) were often detected between the bud scales. **(B)** Representative image of nematodes found associated with the buds at the beginning of winter. **(C-F)** Dead nematodes found within the buds; (C-D) eggs and (E-F) adult females. **(G-I)** Active juvenile nematodes with characteristic dark intestines that are filled with numerous lipid droplets. Immature females are the most common and active stage (I). Scale bars: 200 µm (A, B); 20 µm (C-I).
