# Supplementary material for: Cellular insights of beech leaf disease reveal abnormal ectopic cell division of symptomatic interveinal leaf areas
Source: PLoS One. 2023 Oct 5;18(10):e0292588. doi: 10.1371/journal.pone.0292588 (PMC10553357; doi:10.1371/journal.pone.0292588)
Supplement: S5 Fig — (A-B) Nematodes collected from twigs. (C) Amplification curves obtained from four sets of nematodes collected from independent beech twigs (red). Nematodes collected from buds were used as positive control (blue). Water was used as negative control (orange). Nematode detection was conducted using a fatty acid- and retinol-binding gene (FAR) of L. crenatae subsp. mccannii. Scale bars: 100 μm. (PDF) [file pone.0292588.s012.pdf]

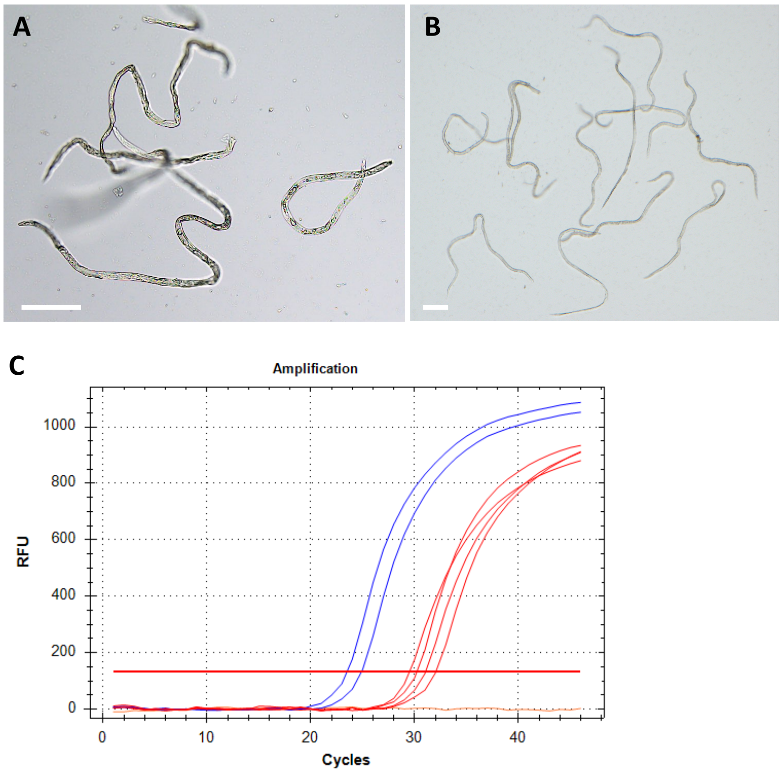

S5 Fig. Detection of *Litylenchus crenatae* subsp. mccannii *collected* from the bark of beech twigs. (A-B) Nematodes collected from twigs. **(C)** Amplification curves obtained from four sets of nematodes collected from independent beech twigs (red). Nematodes collected from buds were used as positive control (blue). Water was used as negative control (orange). Nematode detection was conducted using a fatty acid- and retinol-binding gene (FAR) of L. crenatae subsp. mccannii. Scale bars: 100 µm.
