# Supplementary material for: Cellular insights of beech leaf disease reveal abnormal ectopic cell division of symptomatic interveinal leaf areas
Source: PLoS One. 2023 Oct 5;18(10):e0292588. doi: 10.1371/journal.pone.0292588 (PMC10553357; doi:10.1371/journal.pone.0292588)
Supplement: S6 Fig — (A) Asymptomatic beech leaf collected in early spring. (B-E) Symptomatic BLD leaves collected in early spring showing the typical interveinal green banding. Examples of symptomatic BLD crinkled leaves (D-E). (F) Asymptomatic beech leaf collected in early autumn. (G-J) Symptomatic BLD leaves collected in early autumn showing the typical interveinal green banding with some interveinal areas with yellow and brown stripes. (PDF) [file pone.0292588.s013.pdf]

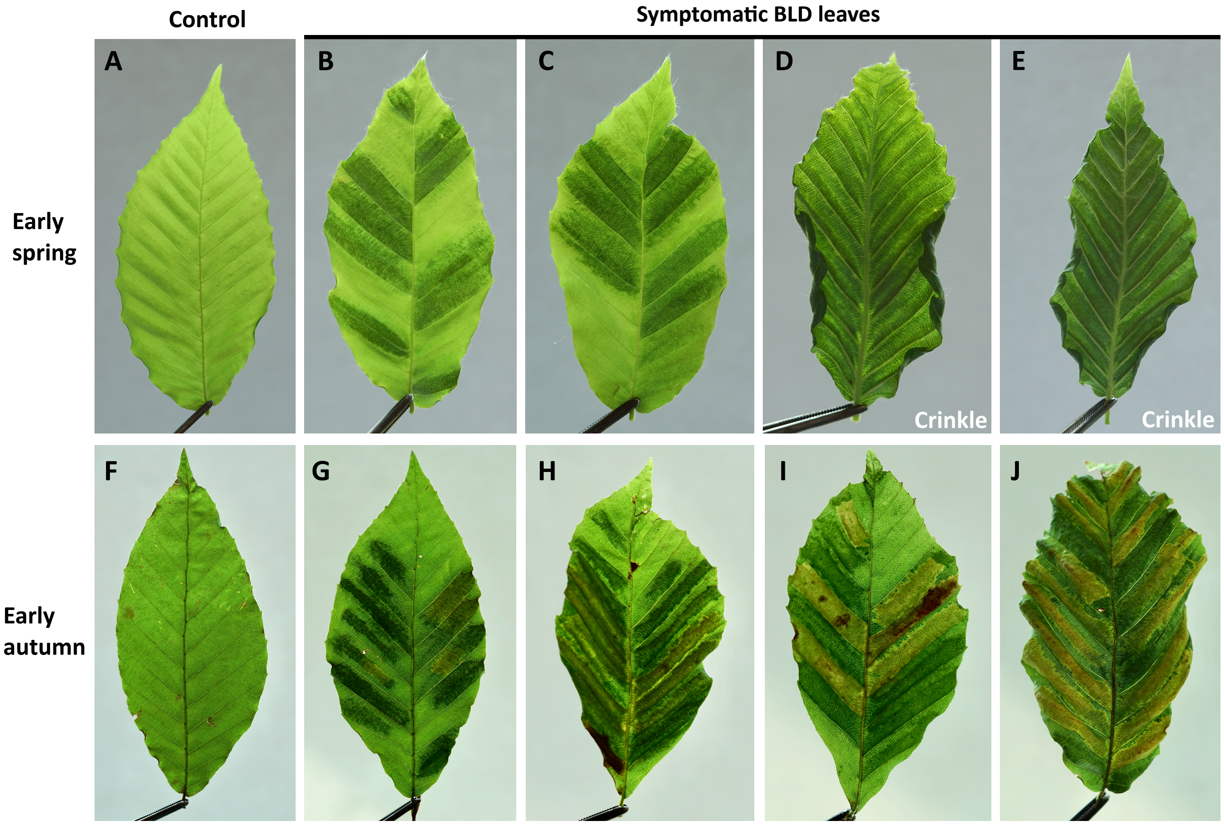

S6 Fig. Representative asymptomatic and symptomatic beech (*Fagus grandifolia*) leaf disease (BLD) leaves collected in the spring and autumn. (A) Asymptomatic beech leaf collected in early spring. **(B-E)** Symptomatic BLD leaves collected in early spring showing the typical interveinal green banding. Examples of symptomatic BLD crinkled leaves (D-E). **(F)** Asymptomatic beech leaf collected in early autumn. **(G-J)** Symptomatic BLD leaves collected in early autumn showing the typical interveinal green banding with some interveinal areas with yellow and brown stripes.
